# From lipid switch to tissue repair: how resolvins reprogram macrophage polarization and function

**DOI:** 10.1097/IN9.0000000000000076

**Published:** 2026-01-30

**Authors:** Rebecca Buete, Jordan Scherer, Andreas Patsalos, Laszlo Nagy

**Affiliations:** 1 Departments of Medicine and Physiology, Pharmacology and Therapeutics and Biomedical Engineering, Johns Hopkins University School of Medicine, Institute for Fundamental Biomedical Research, Johns Hopkins All Children’s Hospital (JHACH), St. Petersburg, FL, USA; 2 Department of Medicine, Johns Hopkins University School of Medicine, Institute for Fundamental Biomedical Research, Johns Hopkins All Children’s Hospital (JHACH), St. Petersburg, FL, USA

**Keywords:** specialized pro-resolving mediators, d-series resolvins, resolution of inflammation, macrophage, macrophage signaling, macrophage polarization, metabolic disease, immunomodulation, atherosclerosis, diabetes mellitus, rheumatoid arthritis, duchenne muscular dystrophy

## Abstract

Specialized pro-resolving mediators (SPMs) derived from docosahexaenoic acid (DHA), particularly D-series resolvins (RvD1, RvD2, RvD3, and RvD5), function to terminate inflammation while preserving host defense. They are synthesized from DHA by lipoxygenases and act through G‑protein‑coupled receptors and lipid‑sensing transcription factors (TFs). These mediators reprogram macrophage metabolism towards fatty‑acid oxidation and oxidative phosphorylation, accelerate efferocytosis, and promote tissue repair. Here, we synthesize current knowledge on their biosynthesis, receptor signaling, and immunometabolic rewiring within macrophages, and critically appraise their therapeutic potential across cardiometabolic, musculoskeletal, autoimmune, and ischemia/reperfusion disorders. We also discuss analytical controversies surrounding their in vivo low‑abundance detection, and outline translational challenges including short half‑life, formulation stability, and emerging synthetic agonists. Finally, we propose priority research directions, from single-cell spatial lipidomics to clinical translation, to define the next frontier for resolvin-based immunotherapies.

## 1. Introduction

**Figure 1.**
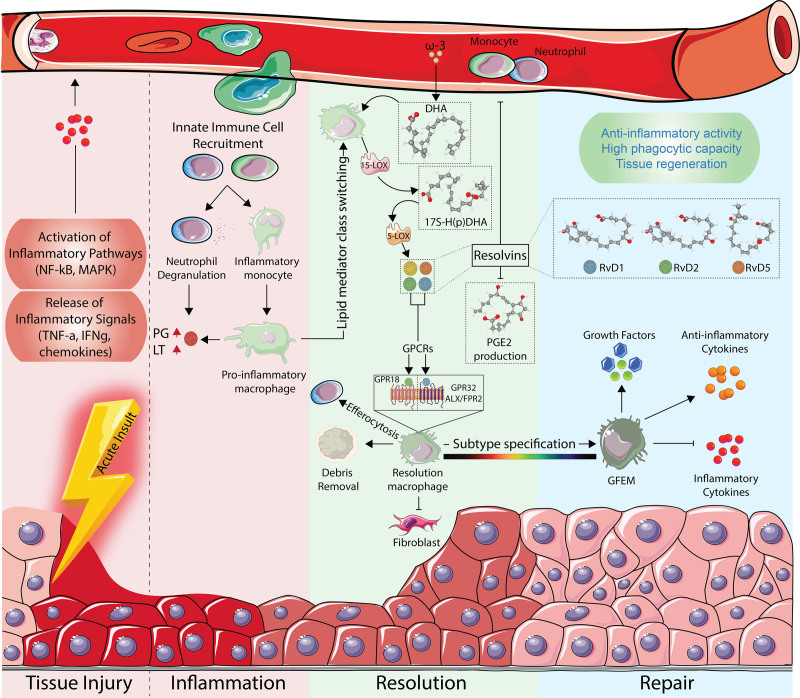
**Initiation and Resolution of Inflammation Mediated by Resolvin Signaling.** Tissue trauma triggers local cells to release cytokines and chemokines, recruiting neutrophils and monocytes and activating pro-inflammatory transcriptional programs. Infiltrating neutrophils degranulate, while monocytes differentiate into Ly6C^hi^ MFs secreting prostaglandins (PGs) and leukotrienes (LTs) into the tissue milieu. As the response shifts toward resolution, these MFs undergo lipid-mediator class switching and adopt a Ly6C^lo^ repair, alternatively activated phenotype. Using 5- and 15-lipoxygenases (5-LOX/15-LOX), these MFs convert DHA from ω-3 fatty acids into DHA-derived resolvins which are secreted into the tissue microenvironment. The resolvins interact with GPCRs on macrophages to reinforce their pro-resolution phenotype. Finally, a subset of these resolution MFs further specialize into growth factor expressing macrophages (GFEMs), whose unique transcriptional profile drives tissue repair and restoration of homeostasis.

Macrophages (MFs) are a highly plastic cell type that are indispensable during the inflammation process and its resolution. Inflammatory signals recruit Ly6C+, CD34^−^ classical myeloid-derived blood monocytes to sites of inflammation, which then differentiate into the classical inflammatory Ly6C^hi^ MFs ^[1]^. However, as inflammation progresses, the microenvironment within the inflamed tissue changes. MFs receive signals that cause an in situ transition to an anti-inflammatory, alternatively polarized, Ly6C^lo^ state ^[2]^. While it was initially thought that inflammation resolves passively as inflammatory signals are degraded, there is now ample evidence to suggest that MFs, in concert with many other cell types, contribute to the active resolution of inflammation. Recently, using spatial transcriptomics, this population of Ly6C^lo^ cells has been dissected, and a new, growth factor-expressing MF was elucidated ^[3]^. These MFs actively secrete growth factors, including Insulin-like growth factor 1 (IGF1), Glycoprotein Non-metastatic Melanoma protein B (GPNMB), Growth/Differentiation Factor-3 (GDF-3), and GDF-15, which contribute to the resolution of inflammation. Additionally, MFs undergo lipid class switching ^[4,5]^. During this process, the production of inflammation-associated lipids, such as prostaglandins and leukotrienes, is downregulated, and omega-3 essential fatty acids, eicosapentaenoic acid (EPA) and docosahexaenoic acid (DHA), are metabolized to produce specialized pro-resolving mediators, including resolvins, protectins, and maresins ^[6,7]^. Maintaining basal levels of inflammatory lipids signals recruits immune cells, while simultaneous upregulation of SPMs can polarize these cells within the tissue microenvironment. SPMs have been shown to decrease neutrophil infiltration, promote efferocytosis, and reduce inflammatory cytokine and chemokine signaling, all of which are essential to the resolution of inflammation (**Figure 1**). This switch represents a key active feedback mechanism to limit inflammation spatially and temporally.

While under normal conditions, inflammation subsides, in many metabolic diseases, such as atherosclerosis, rheumatoid arthritis, diabetes, muscular dystrophy, etc., chronic, consecutive inflammatory events prevent proper return to tissue homeostasis. Inflammatory and anti-inflammatory signals overwhelm the tissue milieu, leading to immune asynchrony, as immune cells cannot properly coordinate the immune response, resulting in tissue damage. Recently, there has been interest in modulating the immune response by using SPMs to promote the resolution phase of the inflammatory process. More specifically, DHA-derived resolvins, RvD1, RvD2, RvD3, and RvD5, have been shown to be synthesized by and affect signaling in MFs through their G protein-coupled receptor (GPCR) targets both in vitro and in vivo. In this review, we aim to outline the synthesis of DHA-derived resolvins and their downstream signaling pathways within the context of MF biology. Furthermore, we examine various metabolic diseases and delineate how resolvins can modulate MF signaling to mitigate chronic inflammation.

While there has been ample evidence to stimulate macrophage, what else would we do if you were not in this lab.

## 2. Resolvin biosynthesis by MFs

d-Series resolvins are synthesized from DHA, an omega-3 fatty acid primarily found in fatty fish or fish oil dietary supplements. Upon consumption, it is incorporated into adipose tissue and cell membranes for long-term storage. During inflammation and its resolution, DHA and other lipid mediators are released from these fat stores, where MFs and other cells can synthesize lipid signaling molecules. MFs express both 15-lipoxygenase (15-LOX) and 5-lipoxygenase (5-LOX), the two enzymes that act sequentially to catabolize DHA, allowing for the complete synthesis of resolvins (**Figure 1**) ^[8,9]^. Additionally, epithelial cells can transcellularly produce resolvins in conjunction with neutrophils. In MFs, 15-LOX stereospecifically oxygenates C17 of DHA, primarily producing 17*S*-HpDHA (rapidly reduced to 17*S*-HDHA) ^[10]^. This intermediate is further oxygenated by 5-LOX to form 7-hydroperoxy-17*S*-HDHA, which is quickly dehydrated to 7(8)-epoxy-17*S*-HDHA. Finally, hydrolysis of the epoxide forms either RvD1 or RvD2, depending on the location of the resulting three hydroxyl groups ^[8,11]^. Alternatively, 7-hydroperoxy-17*S*-HDHA can be reduced to form RvD5. 5-LOX can also oxygenate 17*S*-HDHA at C4, producing 4-hydroperoxy-17*S*-HDHA. Following similar biosynthetic mechanisms, this intermediate undergoes either dehydration to form 4(5)-epoxy-17*S*-HDHA, which can then be rehydrated to produce RvD3 and RvD4, or is reduced to form RvD6. Comprehensive figures summarizing the biosynthetic pathways discussed here are available in previously published articles ^[9–11]^.

While MFs endogenously produce SPMs, resolvin production can be modulated exogenously by aspirin-induced acetylation of cyclooxygenase-2 (COX-2) within the epithelium. Acetylated COX-2 can directly act upon DHA to produce 17R-HpDHA (reduced to 17R-HDHA), the stereoisomer of the 15/5-LOX pathway intermediate ^[12]^. 17R-HDHA can be translocated to both neutrophils and MFs, where it is further metabolized by 5-LOX into epoxide intermediates, which are then hydrated to AT-RvD1, AT-RvD2, AT-RvD3, and AT-RvD4 ^[10]^. These resolvins retain the opposite stereochemistry at C17 but have been shown to have similar in vivo effects compared to their endogenous counterparts ^[9]^. Treating animals with aspirin has been shown to increase the production of AT-resolvins and is associated with enhanced MF efferocytosis ^[13]^. However, AT-resolvins require more thorough investigation of in vivo and in vitro dynamics, including receptor-binding assays and gene-expression/signaling analyses, to assess how AT-resolvins support an anti-inflammatory, pro-regenerative MF phenotype.

## 3. D-Series resolvin signaling alters MF polarization

RvD1 and its GPCR receptors, Lipoxin A_4_ Receptor/Formyl Peptide Receptor (ALX/FPR2) and G-Protein Coupled Receptor 32 (GPR32 in humans) promote an alternatively polarized, anti-inflammatory MF phenotype. Agonism of ALX/Fpr2 (in mice) has been shown to increase MF efferocytosis of invading neutrophils in numerous studies ^[14–17]^. Efficient clearance of neutrophils by MFs from inflamed tissues is critical, as sustained neutrophil infiltration leads to a prolonged inflammatory microenvironment. While the full mechanisms remain to be elucidated, recent work has shown that RvD1 can downregulate inflammatory miRNAs (mir-155-5p, miR146a-5p, and miR148-3p) and Kruppel-like Factor 5 in response to lipopolysaccharide (LPS) ^[16]^. Similarly, pretreatment of MFs in vitro with RvD1 prior to LPS exposure downregulated TL4-MyD88-mediated NF-κB and MAPK signaling ^[18]^. These MFs displayed lower mRNA levels for inflammatory marker genes, including TNFα, IL1b, and COX-2. Lastly, immortalized microglia treated with RvD1 in vitro displayed increased STAT6 phosphorylation in IL4-pretreated macrophages, enhancing the anti-inflammatory response ^[19]^. Importantly, p-STAT6 regulates key anti-inflammatory and antioxidant genes, reducing metabolic stress in the cell.

In vivo treatment of murine disease models has also helped elucidate RvD1-mediated signaling mechanisms. Non-Alcoholic Steatohepatitis (NASH) mice treated with RvD1 showed increased antioxidation capacity through upregulation of the transcription factor Nrf2 and its downstream target genes. In a model of abdominal aortic aneurysms, treatment with RvD1 in a *Fpr2*^−/−^ knockout mouse model enabled the careful dissection of the molecular mechanism underlying repair MF polarization. Treatment with RvD1 modulated cyclic adenosine monophosphate (cAMP) levels by preventing the binding of phagocyte oxidase 47kDa subunit (p47phox) to NADPH oxidase 2 (NOX2) in MFs ^[17]^. Modulations in cAMP levels have previously been associated with the alternatively polarized macrophage phenotype ^[17]^. Additionally, reduced p47phox binding limited the production of reactive oxygen species, thereby making more oxygen bioavailable for other metabolic processes. In response to RvD1, both fatty acid oxidation and oxidative phosphorylation were increased, resulting in higher levels of available ATP to support the metabolic demands of enhanced phagocytic activity of these MFs ^[20]^. These changes in cell metabolic activity are also mediated through AMPK signaling, which is phosphorylated in response to RvD1 treatment ^[20]^. Finally, in a model of acute respiratory distress syndrome, RvD1 was shown to downregulate MAPK14, which in turn reduced the protein expression of S100A8/A9, leading to enhanced phagocytosis and increased self-renewal in tissue-resident alveolar MFs ^[21]^.

While RvD1 can transmit cellular signals via its GPCR targets, it can be brought into the cytosol and act on lipid-sensing TFs to induce changes in gene expression. One of these TFs, peroxisome proliferator-activated receptor gamma (PPARγ), has been shown to play a crucial role in alveolar MF lineage determination, lung surfactant signaling, and is a part of the PPARγ:RXR axis essential for MF-mediated skeletal muscle regenerative inflammation ^[22,23]^. RvD1, in particular, has been shown to reduce NFkB-p65 subunit activation following LPS administration in the lungs ^[24]^. This led to increased MF phagocytosis and resolution of tissue inflammation. In the same study, knockdown of PPARγ resulted in reduced RvD1 signaling, indicating that RvD1 acts at least indirectly through the PPARγ pathway. Additional lung studies have confirmed the finding that RvD1 can be a potent regulator of alveolar inflammation ^[25]^. In summary, RvD1 partially acts through PPARγ to reduce NFkB-p65 activation and translocation to the nucleus, further reducing the inflammatory nature of the transcriptome. Targeting PPARγ in the lungs with resolvins could be a novel therapeutic strategy to modulate the inflammatory response and promote faster resolution.

RvD2 has been shown to play a role in the anti-inflammatory response through its receptor, G-Protein Coupled Receptor 18 (GPR18). Treatment of human MFs with RvD2 attenuated LPS-induced gene expression changes by lowering toll-like receptor 4 (TLR4) expression levels ^[26]^. Additionally, RvD2 was shown to reduce MF exhaustion after LPS stimulation by decreasing NFkB-p65 activation and tumor necrosis factor alpha (TNFα) production ^[27]^. Interestingly, it was demonstrated that RvD2 could also act in conjunction with TLR2 signaling pathways. TLR2 inhibition abolished the effects of RvD2 treatment ^[27]^. These signals reduce the secretion of inflammatory cytokines, such as Interleukin‑1 beta (IL-1β) and IL-8. The RvD2:GPR18 axis was further shown to cause the phosphorylation of CREB and STAT3 TFs in an RvD2-dependent manner ^[28]^. Phosphorylation of regulatory TFs increased MF phagocytic capacity and demonstrated that p-STAT3 was essential for promoting anti-inflammatory MF polarization. Additionally, p-STAT3 has been shown to be translocated from the nucleus to the mitochondria, where it suppresses ROS production ^[29]^. Similarly, treatment of polymicrobial sepsis with RvD2 increased p-STAT3 and cAMP signaling, increasing MF phagocytosis but also reducing oxidative stress ^[28]^. Further exploring the STAT:RvD2 axis, treatment of MFs with RvD2 reduced phosphorylation of STAT1 and NF-κB, both of which are critical transcription factors in the inflammatory response ^[30]^. Finally, in a model of acute muscle injury, treatment with RvD2 shifted the ratio of Ly6C^hi^:Ly6C^lo^ MFs toward a more anti-inflammatory microenvironment ^[31]^. These Ly6C^lo^ MFs are critical for skeletal muscle regeneration and the precise timing of the inflammatory process. Importantly, in contrast to traditional glucocorticoids, resolvins may offer a novel approach to enhancing the immune system’s ability to resolve inflammation without immunosuppression. Overall, treatment with RvD2 supports the resolution of inflammation by decreasing inflammatory cytokines and polarizing the transcriptome toward a more anti-inflammatory state.

Macrophages also endogenously produce RvD3, a GPR32 agonist, which has been shown to increase MF efferocytosis of neutrophils and to stimulate IL-10 signaling ^[32]^. Similarly, AT-RvD3 (17R-RvD3) stimulated MFs displayed increased efferocytosis and phagocytosis in vitro ^[32]^. Furthermore, AT-RvD3 production has been detected in macrophages, and it has been confirmed as a GPR32 agonist. Treatment of murine and human MFs with RvD3 has been shown to reduce expression of inflammatory cytokines, increase MF efferocytosis of dead neutrophils, and promote an anti-inflammatory MF gene expression paradigm ^[8,33–35]^. Additionally, RvD3 has been postulated to be a “late phase” resolvin that is still produced during the late stages of the inflammatory cycle ^[32]^. This has been hypothesized to reduce the expression of pro-inflammatory cytokines and promote the uptake of cellular debris by MFs, thereby preventing consecutive inflammatory events ^[32]^. Further studies have shown that RvD3 treatment reduces the expression of chemokine attractants, such as CXCL1, CCL2, and CCL11, and decreases the secretion of pro-inflammatory cytokines, including TNF-α, IL-6, and IL-1β ^[33,35]^. Similar to RvD2, RvD3 has also been implicated in skeletal muscle inflammation ^[36]^. Treatment of myocytes with RvD3 increased pAMPK levels, which have direct downstream effects that promote autophagy and limit ER stress in vivo. Beyond this study, there has been very little investigation into the mechanism of action of RvD3. Understanding how RvD3 modulates the transcriptome and secretome of MFs could be one route for advancing current immunomodulatory therapies.

Finally, RvD5 can signal via GPR32 (human) and has similar effects to the other described D-series resolvins. RvD5 was shown to upregulate phospholipase D2 (PLD2), which, in turn, increased expression of CD206, a canonical alternatively activated MF marker gene, and coincided with increased clearance of apoptotic neutrophils ^[37]^. Recently, it has also been shown that RvD5 can also allosterically bind to the prostaglandin EP4 receptor. Interestingly, allosteric regulation of this receptor, in tandem with PGE2 treatment, enhances Gs signaling, increasing cAMP levels ^[38]^. Displaying that SPMs can allosterically modulate other signaling pathways to shift their downstream effects toward an anti-inflammatory state provides researchers with a new perspective on inflammatory receptors that could be co-opted to induce protective effects. Additionally, it was shown that RvD5 regulates MF phagocytosis, and its receptor is upregulated in pro-inflammatory MFs to promote the clearance of phagocytic debris, facilitating a successful transition to the resolution phase of inflammation ^[8]^.

DHA-derived resolvins have substantial effects in modulating the resolution of inflammation through affecting MF signaling (**Figure 1**). Through p-STAT3, p-STAT6, Nrf2, and PPARg, Resolvins induce a cascade of anti-inflammatory transcription factors to activate their target genes. Simultaneously, p-STAT3, cAMP, and AMPK all work together to reduce oxidative stress and maintain a homeostatic oxidative environment. In contrast, SPMs reduce phosphorylation of NF-κB and p-STAT1, reducing inflammatory gene activation and allowing for an anti-inflammatory gene program to dominate. While there is ample evidence for the outcomes and effects of Resolvins, the molecular mechanisms and signaling pathways that underlie these effects remain to be fully dissected. Very few studies have used advanced molecular biology techniques or next-generation sequencing to uncover the molecular mechanisms that govern resolvin signaling. Dissecting the global transcriptomic landscape using RNA-seq upon resolvin binding can uncover MF polarization states and identify functional gene clusters. When paired with epigenomic data such as ATAC-seq and ChIP-seq for histone marks, a more complex understanding of how resolvins modulate macrophage gene expression can be gained. However, even if the full mechanisms are not yet understood, resolvins play a critical role in reducing inflammatory cytokines, increasing MF phagocytosis, and increasing neutrophil efferocytosis without limiting MF activation ^[39,40]^. These molecules could serve as the backbone of new therapeutic strategies, allowing MFs to adopt an anti-inflammatory, regenerative phenotype, without the immunosuppressive effects of traditional steroids.

## 4. Resolvins in cardiovascular pathologies–*atherosclerosis, ischemia-reperfusion injury and stroke*

d-series resolvins have emerged as key modulators of macrophage signaling during cardiovascular tissue repair. Atherosclerosis, characterized by chronic vascular inflammation and plaque accumulation, provides a particularly relevant setting. The RvD1 receptor GPR32 co-localizes with CD68^+^ macrophages and the anti-inflammatory marker Arginase-1 ^[41]^. Further, its mRNA levels correlate positively with *Arg1* as well as with M1-associated genes *Il23a* and *Nos2* and the T-reg marker *Foxp3*, highlighting the nuanced role of GPR32 in pro- and anti-inflammatory signaling, and designating it as a potential pro-resolving target ^[41]^. In the same study, transgenic mice that express human GPR32 on an *Apoe*^−/−^ *Fpr2*^−/−^ background (murine model of atherosclerosis combined with Fpr2 knockout; mice do not express GPR32) develop markedly smaller aortic-arch plaques than littermates lacking an RvD1 receptor, consistent with earlier reports that GPR32 activation enhances phagocytosis and suppresses pro-inflammatory cytokine production in atherosclerotic lesions ^[41–43]^. In parallel, 17R-RvD1 has recently been shown to attenuate ER stress in Sickle Cell Disease-associated cardiomyopathy, reducing Unfolded Protein Response (UPR) activation and protecting against maladaptive cardiovascular remodeling after Hypoxia/Reoxygenation (H/R) injury ^[44]^.

**Figure 2.**
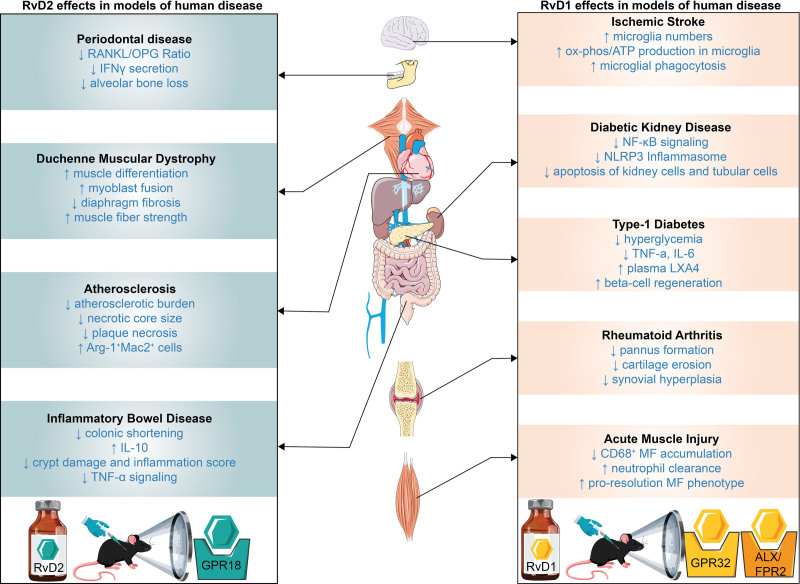
**Multiorgan Effects of Resolvin Based Therapeutics in Diseases.** Resolvins polarize macrophages toward an anti-inflammatory program, and they have also been shown to be effective in treating various mouse models of human diseases. This schematic outlines the effects of specific resolvin treatment on different disease models. On the right, the models were treated with RvD1 while the models on the left were treated with RvD2. All treatments were performed via intraperitoneal or via tail vain injection with the dosing scheme as follows: Atherosclerosis (4 weeks, 3i.p./week, 100 ng), Inflammatory Bowel Disease (4 days, daily i.p., 0.3 μg or 1 μg) Rheumatoid Arthritis (48 days, tail vain Injection every third day, 20 ng or 100 ng), Stroke (1, 3, or 7 days, daily i.p., 15mg/kg), Type 1 Diabetes (5 days, daily i.p., 60ng), Periodontal Disease (2 weeks, every other day, 0.5 μg on first dose followed by 0.1ug on subsequent doses), Diabetic Kidney Disease (i.p. 30 minutes prior to injury, 50 μg/kg), Acute Muscle Injury (i.p. 5 minutes prior to injury, 100 ng), Duchenne Muscular Dystrophy (1 or 3 weeks, daily or weekly i.p., 5 μg/kg). The disease specific outcomes for each treatment are summarized in the corresponding boxes. Abbreviations: Receptor Activator of Nuclear Factor кB Ligand/ Osteoprotegerin (RANKL/OPG), Interferon gamma (IFNγ), Arginase1 (*Arg1*), Galectin-3 (*Mac2*), NOD‑, LRR‑ and pyrin domain‑containing protein 3 (NLRP3), Lipoxin A_4_ (LXA_4_).

Attention has likewise turned to the RvD2-GPR18 axis as a potential therapeutic target for its atheroprotective activity, after the pair was detected in human coronary arteries in relation to atherosclerotic lesions ^[45]^. In *Apoe*^−/−^ mice, exogenous RvD2 reduces atherosclerotic burden and necrotic-core size, effects that are lost when GPR18, expressed by myeloid cells ^[46]^, is blocked with the antagonist O-1918 ^[45]^. Another study utilizing a myeloid-specific *Gpr18* deletion in *Ldlr*^−/−^ mice exaggerates plaque necrosis, elevates cleaved-caspase-3, and increases the number of pro-inflammatory CD68^+^ macrophages, thereby rendering lesions unresponsive to RvD2. Conversely, a humanized GPR18 knock-in preserves the atheroprotective response ^[47]^. Complementing these receptor-focused studies, MLKL-deficient, PCSK9-mutant mice exhibit higher tissue RvD1/17-HDHA levels, diminished prostaglandins, improved plaque resolution, and enhanced macrophage oxidative phosphorylation and ATP synthesis, all of which accelerate clearance of necroptotic debris ^[20]^. Together, these data position resolvin-GPCR pairs as tractable targets that restore pro-resolution programs rather than simply suppressing inflammation, distinguishing them mechanistically from NSAIDs or corticosteroids (**Figure 2**).

Ischemia-reperfusion injury and stroke offer additional contexts in which resolvins recalibrate immune energetics ^[48,49]^. Endogenous resolvin production falls sharply after cerebral ischemia ^[50]^, yet intraperitoneal administration of RvD1 post-stroke boosts microglial numbers, increases neutrophil efferocytosis on days 1 and 3, and reprograms microglial metabolism from glycolysis to oxidative phosphorylation, thereby expanding ATP reserves needed for phagocytosis ^[51]^. Collectively, these findings highlight a unifying theme: resolvins engage specific GPCRs to shift macrophages and microglia toward a reparative, metabolically efficient phenotype, curbing chronic inflammation and fostering tissue restoration across atherosclerosis, ischemic heart disease, and neurovascular injury.

## 5. Metabolic disease pathophysiology—*diabetes mellitus*

In line with findings by Hosseini and colleagues ^[20]^, attenuation of mitochondrial dysfunction through resolvin-mediated tissue regeneration is increasingly understood to be beneficial for cellular bioenergetics (ATP production). In Fat-1 mice, which endogenously convert omega-6 fatty acids into omega-3 polyunsaturated fatty acids, the lipidome is enriched in RvD1, and the animals are protected from TNF-α-induced mitochondrial dysfunction, which helps explain why omega-3 enrichment lowers metabolic-disease risk ^[52]^.

A parallel emerges in inflammatory bowel disease, where mice with colitis exhibit a plasma deficit of RvD2. Replenishing this mediator, either by intraperitoneal injection or omega-3 feeding, reduces colonic shortening and inflammation as effectively as anti-TNF therapy, without systemic immunosuppression (**Figure 2**) ^[53]^. Unsurprisingly, RvD2 likewise dampens TNF-α signaling in other tissues ^[54]^, illustrating how SPMs can substitute for, or augment cytokine-targeted drugs. Overall, attenuation of chronic inflammation through administration of resolvins or their precursors not only modulates leukocyte gene expression and cytokine signaling but also supports efficient MF bioenergetics.

In diabetes and diabetic wound healing, characterized by persistent scarring and lack of angiogenesis ^[55]^, SPMs intervene at several pathological nodes. In streptozotocin-induced type 1 diabetic mice, RvD1 lowers hyperglycemia and inflammation, an effect partially attributed to downstream restoration of lipoxin A_4_ secretion ^[56]^. In the context of repair, RvD1 improves renal outcomes after ischemia-reperfusion in diabetic mice by blocking NF-кB activation through inhibition of IкBα and p65 phosphorylation ^[57]^. In parallel, hyperglycemia-driven periodontal disease in type 2 diabetes is attenuated when RvD1 reduces the RANKL/OPG ratio and IFN-γ secretion, thereby limiting alveolar bone loss ^[58,59]^. A few studies also demonstrate that resolvin treatment accelerates re-epithelialization and closure of cutaneous wounds ^[60–63]^, although its efficacy in overtly diabetic skin remains underexplored. Resolvins and related SPMs are therefore positioned as promising adjuncts for chronic metabolic disorders through counteracting TNF-α-centered inflammation, preserving mitochondrial function, and enhancing tissue repair across multiple diabetic complications (**Figure 2**).

## 6. Autoimmune disease—*rheumatoid arthritis*

Elevated plasma SPMs correlate with clinical improvement in rheumatoid arthritis (RA) and may predict response to therapy ^[64,65]^. In a metabolomic study, concentrations of the DHA metabolome (including d-series resolvins, protectins, and maresins) were measured by LC-MS/MS in RA patient serum. This predicted disease-modifying antirheumatic drug (DMARD) responsiveness with 81% accuracy, driven largely by selective up-regulation of RvD4 in non-responders ^[66]^. Thus, RvD4 emerged as both a pathotype marker and a potential therapeutic target for limiting joint damage. Moreover, in collagen-induced arthritic mice, RvD1 dose-dependently reduced pannus formation, cartilage erosion, and synovial hyperplasia on H&E/toluidine blue sections ^[67]^. Interestingly, these structural benefits paralleled lower serum levels of connective tissue growth factor (CTGF), IL-6, IL-1β, and TNF-α. Sun et al traced these effects to RvD1-mediated induction of miR-146 and subsequent suppression of CTGF, ultimately ameliorating RA progression. Collectively, the findings align with epidemiological evidence that omega-3-rich diets reduce RA severity by boosting endogenous SPM production ^[68]^, suggesting the use of SPMs as both biomarkers and effectors of disease control. (**Figure 2**).

## 7. Musculoskeletal pathologies—duchenne muscular dystrophy and other muscle repair dysfunction

Immune modulation and proper leukocyte function are central to skeletal-muscle regeneration. SPMs such as lipoxins and resolvins and their precursors act through ligand-activated TFs to temper inflammation and guide repair ^[4]^. In 12/15-LOX-null mice that are unable to generate many resolvins, muscle injury triggers excessive leukocyte influx and an exaggerated acute inflammatory response compared to wild-type models ^[69]^. Although the short (hour-range) half-life of SPMs complicates mechanistic studies ^[58,70]^, exogenous SPM delivery remains a promising strategy for diseases marked by impaired muscle repair.

In a model of acute, sterile injury with cardiotoxin (CTX), pro-inflammatory lipid mediators decrease, while SPMs rise between days 2 and 8 ^[31]^. RvD2 and RvD5, along with their pathway markers, remained elevated 4 days post-injury as compared to baseline uninjured skeletal muscle ^[31]^. In eccentric injury, the same DHA-derived resolvins peak only at day 8 ^[31]^. Importantly, distinct lipid mediator profiles of Ly6C^hi/lo^ MFs appeared at day 4 post-CTX injury, representing inflammatory and anti-inflammatory profiles, respectively. Notably, *Gpr18* was expressed in both Ly6C^hi^ and Ly6C^lo^ MFs, whereas RvD2 was primarily produced by Ly6C^lo^ MFs ^[31]^. Consistent with these data, a single intraperitoneal dose of RvD1 at the time of barium chloride (BaCl_2_)-induced muscle injury limited CD68^+^ MF accumulation without affecting neutrophils at 24 hours after injury ^[71]^. Daily RvD1 administration further reduced neutrophil numbers by 40% while leaving MF abundance unchanged ^[71]^. These results align with the findings of Giannakis et al., in which higher intramuscular SPM levels coincide with faster neutrophil clearance and an MF shift toward a pro-resolution state ^[31]^. Modulating this shift in phenotype with SPMs is a potential strategy to curb immune asynchrony observed in musculoskeletal pathologies such as Duchenne Muscular Dystrophy (DMD).

Dort et al. recently showed that RvD2 directly engages GPR18 to promote myogenesis and restore regeneration in DMD (*mdx* muscle) ^[72,73]^. Musculoskeletal pathologies are commonly associated with inflammation, largely mediated by cells of monocytic origin. In vitro, Dort and colleagues assessed the transcriptional profile of monocyte-derived MFs using a standard inflammatory polarization model. IFN-γ/LPS-polarized bone marrow-derived macrophages displayed a pro-resolutive shift following exposure to RvD2, doubling the proportion of Arg1^+^CD206^+^CD163^+^F4/80^+^ cells, lowering iNOS, upregulating PPARγ, CD163, and Anxa1, and downregulating CD80 and GPR18 ^[72]^. In *mdx* satellite cells, RvD2 treatment rescued differentiation, fusion, and myotube growth and did not substantially impact cell proliferation, whereas prednisone inhibited cell expansion ^[72]^. In vivo, RvD2 treatment increased nascent myofibers, reduced diaphragm fibrosis, and improved muscle strength compared to prednisone (**Figure 2**). Confirming the receptor’s sufficiency, a synthetic GPR18 agonist, PSB-KD107, replicated these benefits with 2.3-fold more eMyHC^+^ fibers, larger tibialis anterior fibers, 45% fewer Ly6G^+^ neutrophils, nearly three-fold more CD206^+^ macrophages, and induction of lipid-metabolism genes, which correlates with previously observed anti-inflammatory phenotypes in myoblasts ^[73]^. Together, these findings establish GPR18 activation as a discrete, druggable driver of the pro-regenerative, anti-inflammatory program initiated by RvD2 in DMD ^[72,73]^.

A more recent study by Markworth and Brown linked chronic myofiber inflammation in aged mice to a deficiency of intramuscular RvD1 ^[74]^. Aged tibialis anterior muscles showed lower baseline SPMs and precursors than young muscle. Following BaCl_2_ injury, RvD1 supplementation did not change macrophage or neutrophil cell counts at day 3 but increased the number of actin^+^ contractile fibers and improved functional myogenesis without hypertrophy. Nevertheless, aged muscles still formed fewer and smaller eMyHC^+^ fibers at day 5 post-injury than younger animals, underscoring persistent age-related constraints.

These findings highlight dysregulated lipid-mediator balance as a driver of chronic inflammation in aging, and support SPM delivery as a potential therapy for other conditions such as DMD or cachexia, where repair is often derailed by fibrosis, adiposity, or maladaptive hypertrophy.

## 8. Current advances in resolvin-based therapeutics

Currently, there has been some interest to develop Resolvin-based therapeutics; however, manufacturing and stability concerns have derailed development. Revolvins and other lipid mediators have extremely short half-lives, are light sensitive, and can degrade quickly at room temperature. Companies have begun to develop chemically stabilized resolvin analogues, such as the DPA-derived RvE1 pro-drug RX-10045, which completed placebo-controlled Phase II trials for dry eye disease and demonstrated safety and target engagement in more than 200 patients ^[75]^. Nanoparticle, hydrogel, and micellar delivery systems (~12 nm micelles used for RX-10045) remain stable for a week at room temperature, thereby extending the half-life, increasing aqueous stability, and mitigating the pharmacokinetic limitations of SPM-based drugs. However, RX-10045 clinical trials were unceremoniously discontinued when results with and without treatment were found to be equivocal ^[75]^. Furthermore, receptor-selective small-molecule agonists, such as the GPR18 ligand PSB-KD107, now in pre-clinical development for Duchenne muscular dystrophy, show that non-lipid mimetics can retain pro-resolving efficacy while resisting degradation ^[73]^. The anti-inflammatory effects of RvD2, we predict, may exert long-term effects in dystrophic muscle. Finally, plasma and lesional SPM signatures already predict DMARD responsiveness in rheumatoid arthritis with approximately 80% accuracy, and deeper integration of spatial lipidomics with single-cell omics could enable patient stratification and dose selection in forthcoming trials ^[66]^. These advances collectively indicate that resolvin-based therapeutics are progressing beyond preclinical, proof-of-concept drugs toward clinically actionable interventions that modulate inflammation without overt immunosuppression while preserving host defense.

## 9. Methodological controversies in SPM detection

Recently, controversy in the SPM field has centered on the reliability and reproducibility of lipid-based measurements and datasets. Debate has arisen over how best to detect specialized pro-resolving mediators (SPMs), with a focus on liquid chromatography-tandem mass spectrometry (LC-MS/MS) criteria. O’Donnell et al re-examined the plasma lipid-omics dataset of Gómez et al ^[66]^. and argued that applying a more conservative lower limit of quantification (LLOQ), defined as signal-to-noise (S/N) > 5, eliminates many putative SPM peaks, implying that these mediators are rarely detectable in vivo ^[76]^. Gómez and Dalli countered, stating that multiple industry-accepted standards already exist for setting the LLOQ, showed that re-analysis with an S/N > 5 replicated their original metabolite calls, and noted that several authors of the critique have themselves published quantitative SPM measurements in diverse tissues ^[77]^. Despite the disagreement, both groups endorse rigorous, standardized workflows. O’Donnell’s team continues to report *n*-3 PUFA-driven macrophage reprogramming and endogenous SPM generation in metabolic and inflammatory contexts ^[78–80]^, while cautioning that resolvins production may be limited under basal conditions ^[81]^. They have recently proposed detailed LC-MS/MS guidelines for oxylipin analysis ^[82]^. Addtionally, detection of resolvins via commercial ELISA kits is available, but low in vivo abundance, structural isomer detection, and potential matrix effects maintain LC-MS/MS as the gold standard for resolving detection. For GPCR detection, few ELISAs are validated, and the standard of detection includes IHC/Flow cytometry, targeted proteomics, or mRNA detection via qPCR. Regardless of SPMs lying at or below present detection thresholds, synthetic agonists and exogenous SPM administration reproducibly modulate inflammatory circuits, and a wider lipidomic view underscores the central role of omega-3-derived mediators in coordinating cells, cytokines, and growth factors during tissue repair ^[83]^. Thus, refining analytical standards should proceed in parallel with functional studies that exploit these potent immunoresolvents.

## 10. Conclusions

SPMs, and more specifically, DHA-derived resolvins are potent regulators of leukocyte phenotype, cytokine expression, and the overall progression and resolution of inflammation. By imbuing leukocytes with an anti-inflammatory toolkit and fostering tissue repair, resolvins occupy a unique therapeutic space in diseases where chronic or pro-oxidative inflammation has been shown to impair regenerative processes. Extending our knowledge of resolvin signaling to different tissue types, especially skeletal muscle, represents a promising avenue to broaden their therapeutic impact. Uncovering new ligand-receptor interactions and downstream signaling pathways will deepen our mechanistic understanding of the SPM axis and inform targeted therapies for inflammatory diseases and cancer. Recent investigations are beginning to elucidate how malignant cells co-opt resolution pathways, an emerging frontier in SPM research. Improving our understanding of the temporal and spatial regulation of resolvin signaling and their integration with existing anti-inflammatory therapies will be critical for the development and successful introduction of SPM-based therapeutics for chronic inflammatory diseases.

## Conflict of interest

The authors have no conflict of interest.

## Funding

5R01DK124782-04 DHA-derived resolvin production and signaling in tissue repair macrophages in metabolic disease.This work was supported by an MDA Development Grant (#1064247) to A.P. and NIH DK115924, AI 185363 and HL170426 to L.N.

## Acknowledgements

RB and JS wrote the paper; AP and RB designed the figures; AP and LN had primary responsibility for final content. RB, JS, and AP revised the manuscript. All authors read and approved the final manuscript.

## Additional Information

Figures were generated using Adobe Illustrator 29.6.1. Structures generated using MolView. Image(s) provided by Servier Medical Art (https://smart.servier.com), licensed under CC BY 4.0.
